# WBT-DC pipeline: a cross-cohort and cross-platform disease classification pipeline based on whole-blood transcriptomics

**DOI:** 10.1186/s12967-026-08254-3

**Published:** 2026-05-11

**Authors:** Mengzhen Li, Han Jin, Lingqi Meng, Ozlem Altay, Bayram Yuksel, Cheng Zhang, Mathias Uhlen, Hasan Turkez, Adil Mardinoglu

**Affiliations:** 1 Science for Life Laboratory, KTH – Royal Institute of Technology, Stockholm, SE-17165 Sweden; 2 SZA Omics R&D, Mehmet Ali Aydinlar Acibadem University, Istanbul, Türkiye; 3 Liver Institute, King’s College London, London, UK; 4 Department of Medical Biology, Faculty of Medicine, Atatürk University, Erzurum, 25240 Türkiye; 5 Centre for Host-Microbiome Interactions, Faculty of Dentistry, Oral & Craniofacial Sciences, King’s College London, London, SE1 9RT UK

**Keywords:** Whole blood transcriptomics, Machine learning, Disease prediction

## Abstract

**Background:**

Machine-learning models based on tissue transcriptomic data are powerful tools for disease classification. However, their clinical adoption is limited by the invasive nature of tissue sampling. Furthermore, transcriptomic datasets are often affected by batch effects and gene-level noise, which compromise model generalizability across platforms and clinical cohorts.

**Methods:**

We developed WBT-DC (Whole Blood Transcriptomics–based Disease Classification), a computational pipeline designed to overcome these challenges. WBT-DC integrates rank-based feature extraction to mitigate batch effects with an ensemble machine-learning framework that incorporates cross-validation and hyperparameter optimization. Its performance was systematically evaluated across five independent cohorts involving 2,164 participants and three disease contexts: Crohn’s disease (CD), ulcerative colitis (UC), and amyotrophic lateral sclerosis (ALS). We tested the model’s robustness across RNA-sequencing and microarray platforms. Additionally, an internal rheumatoid arthritis (RA) cohort (*n* = 165) was utilized for real-world prospective validation.

**Results:**

WBT-DC demonstrated high accuracy, achieving ROC–AUC values of 0.90–0.94 in independent datasets when training and testing were conducted on the same platform. In cross-platform evaluations, the pipeline maintained robust performance with ROC–AUC values ranging from 0.71 to 0.84, consistently outperforming conventional gene expression-based models. In the RA validation cohort, WBT-DC achieved an ROC–AUC of 0.81, supporting its applicability in a real-world clinical setting.

**Conclusions:**

WBT-DC provides a robust, non-invasive, and platform-agnostic framework for disease classification using whole-blood transcriptomics. By effectively addressing batch effects and platform variability, this pipeline offers a scalable solution for translating systems-level transcriptomic insights into applications.

**Supplementary Information:**

The online version contains supplementary material available at 10.1186/s12967-026-08254-3.

## Graphical Abstract

**Supplementary Information:**

The online version contains supplementary material available at 10.1186/s12967-026-08254-3.

## Graphical abstract

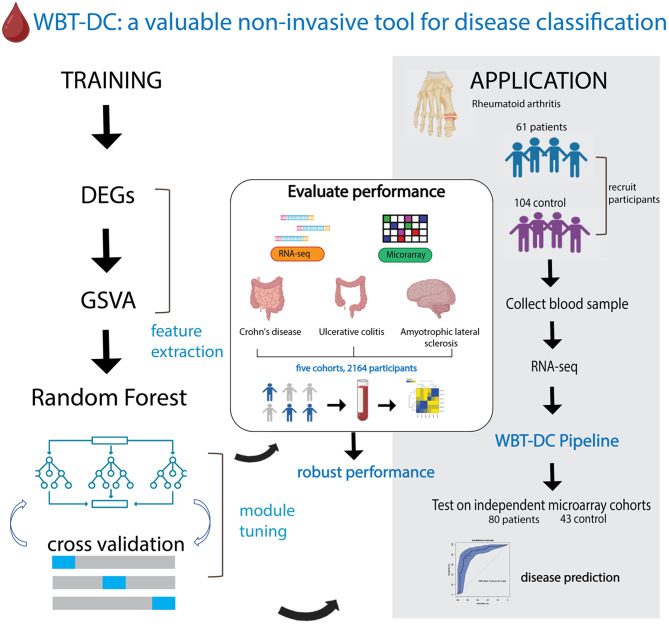

## Introduction

Complex diseases are characterized by transcriptional dysregulation across multiple tissues [1]. As a result, gene expression data from cells and tissues offer valuable insights into disease occurrence and progression. It provides an entry point for characterizing genome-wide functional activity, which is essential for understanding biological processes and elucidating the molecular mechanisms underlying disease development [2]. However, acquiring transcriptomic data from many human tissues is often infeasible (e.g., brain) or requires invasive procedures (e.g., liver and muscle), limiting its widespread clinical application [3].

Systemic disturbances across cells and tissues during disease onset and progression can be reflected in blood [4], making it an attractive medium for monitoring human health. Whole-blood transcriptomic (WBT) data capture the total RNA present in peripheral blood, primarily reflecting immune cell populations such as neutrophils and lymphocytes. Because erythroid cells constitute a large proportion of blood and contribute abundant globin RNA, globin depletion approaches are typically applied to enhance the informative content of the data [5].

Disease classification using WBT data is attractive because of its non-invasive nature and broad molecular coverage [6]. However, extracting informative gene features from the inherently noisy WBT environment remains challenging. Moreover, batch effects intrinsic to transcriptomic data substantially limit model generalizability [7]. As a result, machine-learning models trained on gene expression features often exhibit reduced predictive performance when evaluated on independent cohorts, particularly across different transcriptomic platforms such as RNA sequencing (RNA-seq) and microarrays. Together, these limitations hinder the large-scale deployment of machine-learning–assisted disease classification based on transcriptomic signatures.

In this study, we developed WBT-DC (Whole-Blood Transcriptomics–based Disease Classification), a machine-learning pipeline designed to perform robustly across heterogeneous datasets while minimizing batch effects arising from differences in cohorts and transcriptomic platforms. We first evaluated WBT-DC using publicly available datasets encompassing three diseases—Crohn’s disease (CD), ulcerative colitis (UC), and amyotrophic lateral sclerosis (ALS). Model performance was assessed under both within-platform and cross-platform settings, including RNA sequencing (RNA-seq), Illumina microarrays, and Affymetrix microarrays. We subsequently applied WBT-DC to an in-house rheumatoid arthritis (RA) RNA-seq training cohort and validated its performance using an independent Affymetrix microarray dataset.

## Methods

### Sample collection of rheumatoid arthritis (RA) patients

Ethical approval for this study was obtained from the Clinical Research Ethics Committee of the Medical Faculty at Atatürk University, Erzurum, Turkey (Approval No. 45; 30 December 2021).

For the in-house RA cohort, whole-blood samples were collected at Atatürk University Research Hospital (Erzurum, Turkey) between 20 February 2023 and 25 October 2023. All participants were fully informed about the study, and written informed consent was obtained prior to sample collection. Peripheral whole blood (2–3 mL) was collected into PAXgene Blood RNA tubes (BD Biosciences) containing an RNA-stabilizing reagent.

### Isolation of total RNA from blood samples

Automated RNA isolation was performed using the PAXgene QIAsymphony Blood RNA Kit. RNA quantity and purity were assessed spectrophotometrically using a NanoDrop instrument (Thermo Fisher Scientific, USA). Samples with an A260/280 ratio of approximately 2.0 and an A260/230 ratio between 2.0 and 2.2 were considered acceptable. RNA concentrations were calculated based on OD260 values. RNA integrity was evaluated using a TapeStation system (Agilent Technologies), and samples with an RNA integrity number (RIN) ≥ 5 were deemed suitable for downstream analysis.

### NGS library construction and sequencing

NGS libraries were constructed using the Illumina Stranded Total RNA Prep, Ligation with Ribo-Zero Plus Kit from RNA samples that met the specified quality criteria. Briefly, 100 ng of total RNA was used as input for library preparation.

Following library construction, fragment size distribution and concentration were assessed. Libraries were then normalized and diluted to a loading concentration of 0.4 nM. Sequencing was performed on the Illumina NovaSeq 6000 platform using a paired-end 200-cycle configuration at SZA Omics Labs (Istanbul, Turkey). Raw sequencing data were converted from BCL to FASTQ format and demultiplexed using DRAGEN (v4.2.7).

### Acquisition of publicly available data

We conducted a comprehensive search for publicly available RNA-seq and microarray data on whole blood transcriptomics data in the NCBI Gene Expression Omnibus (GEO) database (https://www.ncbi.nlm.nih.gov/geo/) and the Biostudies dataset (https://www.ebi.ac.uk/biostudies/). Ultimately, we included three CD datasets (GSE186507, GSE112057, GSE119600), three UC datasets (GSE186507, GSE112057, GSE119600), two ALS datasets (GSE112676, E-TABM-940), and one RA dataset (GSE93272). Since the RA cohort (GSE93272) included both treated and untreated RA patients (methotrexate, infliximab, and tocilizumab), only untreated RA patients and healthy controls were included to ensure data homogeneity.

### RNA sequencing data preprocessing

For the RNA sequencing datasets from the public database, the SRA files of each dataset were downloaded from the GEO database using the SRA toolkit and were subsequently converted into raw FASTQ files. Then, Gene expression count data were quantified following the standard protocol of Kallisto (v0.48.0) [8]. The reference cDNA for alignment and quantification was GRCh38 v109 for Homo sapiens, which was retrieved from the Ensemble website (https://www.ensembl.org/index.html). After filtering out non-protein-coding genes and genes with a total count of zero, the remaining gene count data were used for downstream analysis. This resulted in 19,297 protein-coding genes in GSE186507, 17,609 in GSE112057, and 18,537 in the internal RA cohort.

### Microarray data preprocessing

For microarray datasets generated on the Affymetrix platform, raw data were processed from CDF files using the R package affy (v1.80.0) [9]. For Illumina-based datasets, data were processed using the limma package (v3.58.1) [10]. Data preprocessing included background correction, quantile normalization, and log transformation. Non-protein-coding genes were excluded from downstream analyses. For genes represented by multiple probes, the probe with the highest average expression was retained. To reduce noise, genes with an interquartile range (IQR) within the lowest 25% were filtered out. After preprocessing, 12,365 protein-coding genes were retained in GSE119600, 10,343 in both GSE112676 and E-TABM-940, and 12,970 in GSE93272.

### Removal of red blood cell genes

We analyzed the data from a single-cell RNA-seq study of circulating red cells (GSE184916) [11], and found that three genes, HBB, HBA2, and HBA1, were expressed at markedly higher levels than all others. Specifically, their mean expression levels were 113 to 405 times higher than that of the fourth most highly expressed gene (Supplementary Fig. 1). To reduce potential bias and noise, these three genes were excluded from all datasets before downstream analyses.

### Gene expression landscape

#### Dimensionality reduction

To visualize the sample distribution, we performed dimensionality reduction on z-normalized gene expression data. Principal component analysis (PCA) was conducted using the R package pcaMethods (v1.92.0) [12], with the number of principal components selected to explain at least 90% of the total variance. Subsequently, Uniform Manifold Approximation and Projection (UMAP) analysis [13] was performed using the R package uwot (v0.2.2) to project these principal components into two dimensions for visualization.

#### Batch correction

Following the original analysis of the GSE112676 dataset, a batch effect was identified, characterized by differences in IQR, median intensity, and the number of detected protein-coding genes between the first set of 448 samples and the second set of 293 samples, as shown in Supplementary Fig. 2A. To correct this internal batch effect, we applied the ComBat algorithm [14], the same strategy used in a previous study on this dataset [15]. This approach removed the apparent batch effect as demonstrated in PCA plots (Supplementary Fig. 2B).

#### Differential gene expression analysis (DEGs)

For differential gene expression analysis (DEGs), the DESeq2 R package (v1.36.0) [16] was applied to RNA sequencing data, and the limma package was used for the microarray data. The Benjamini-Hochberg (BH) correction was used for multiple testing corrections. Significance for up-regulated genes was determined by an adjusted P-value < 0.05 and a log2 fold change > 0, and for down-regulated genes by an adjusted P-value < 0.05 and a log2 fold change < 0.

#### Pathway enrichment analysis

Gene set overrepresentation analysis (GSOA) was conducted to assess whether known biological functions were enriched among the differentially expressed genes (DEGs) between disease and healthy groups. The identified DEGs were used as the gene set of interest, while all detected genes were defined as the background. GSOA was conducted using the R package clusterProfiler (v4.4.4) [17].

### Machine learning classification

**Fig. 1:**
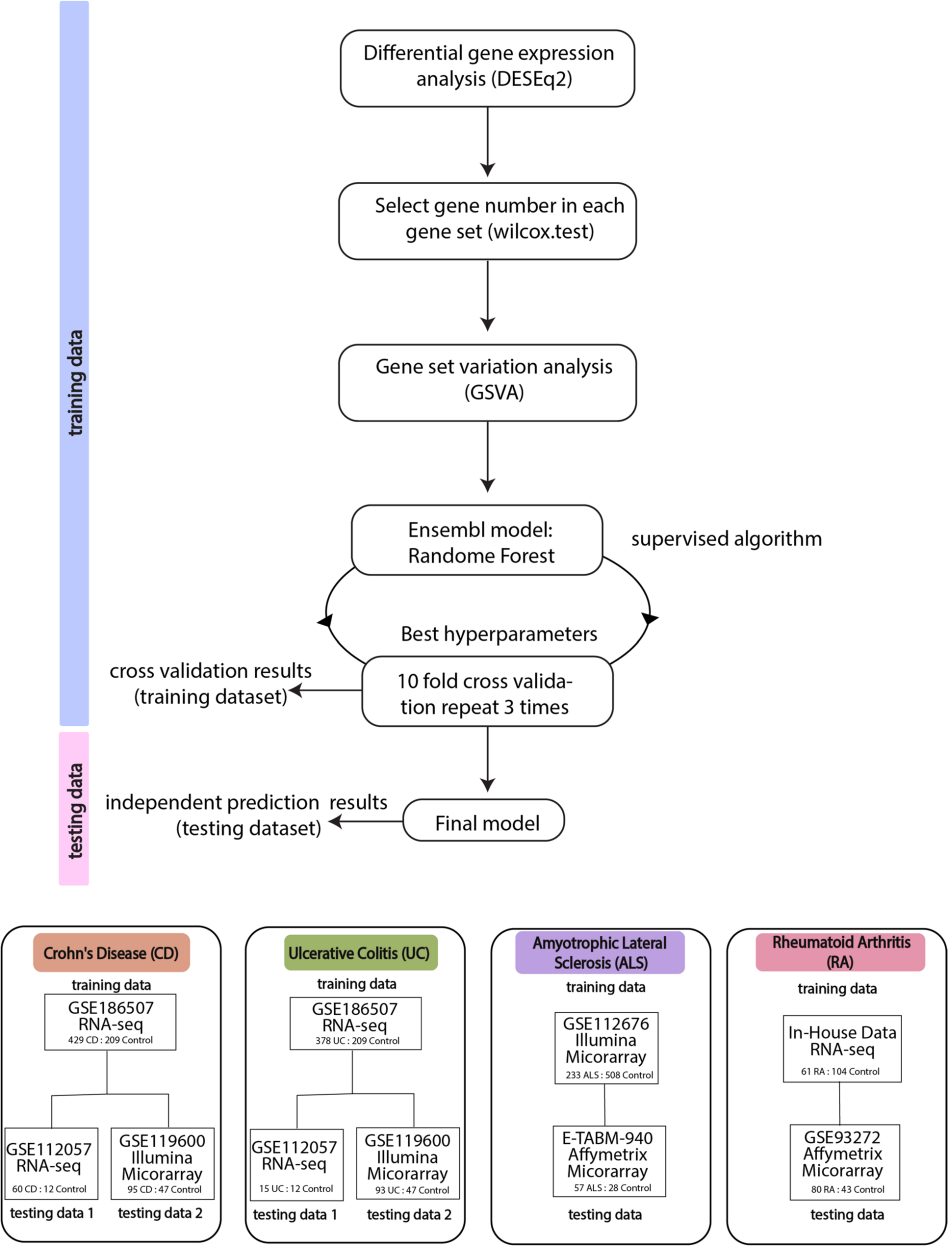
Datasets and workflow of the machine-learning–based WBT-DC pipeline

The WBT-DC pipeline is summarized in Fig. 1. Briefly, gene expression profiles were transformed into gene set enrichment scores using Gene Set Variation Analysis (GSVA), a rank-based enrichment strategy. The resulting enrichment scores were subsequently used as input features for Random Forest (RF)-based disease classification models, followed by systematic hyperparameter tuning to optimize model performance.

#### Feature extraction

To mitigate the batch effect caused by different platforms, we employed the GSVA algorithm [18] to convert gene expression data to gene set enrichment scores, which were then used as features for machine learning. Briefly, GSVA performs kernel estimation of the cumulative density function (kcdf) for each gene within a sample, ranks genes accordingly, and computes a Kolmogorov–Smirnov–like statistic for each predefined gene set based on the ranked gene list. This procedure yields an enrichment score for each gene set in each sample.

The GSVA analysis requires two inputs: predefined gene sets and a gene expression matrix. In this study, predefined gene sets were constructed using randomly selected DEGs identified from the training data by comparing disease and healthy samples. To determine the optimal gene set size, we first assessed the distribution of GSVA scores using the Shapiro–Wilk test and confirmed non-normality. We then applied the Wilcoxon rank-sum test to evaluate differences in enrichment scores between disease and control samples across multiple gene set size intervals. In total, 24 intervals ranging from 10 to 1,000 genes were evaluated. For each interval, the procedure was repeated 1,000 times, including 500 iterations using upregulated genes and 500 using downregulated genes, to ensure robustness.

After identifying the optimal gene set size, gene sets were randomly sampled from the top-ranked DEGs, ordered by the absolute value of their log2 fold change. A total of 200 gene sets were generated, comprising 100 upregulated and 100 downregulated sets, which were used as predefined inputs for GSVA.

For RNA-sequencing data, gene expression matrices were derived from count data following variance-stabilizing transformation (VST). For microarray datasets, log₂-transformed expression values were used. To improve model generalizability, class imbalance in the training datasets was addressed using oversampling with the ROSE package (v0.0-4). Oversampling was applied exclusively to the training data, while the original sample distribution was retained in the testing datasets to reflect real-world conditions. GSVA enrichment scores were calculated using a Gaussian kernel for the kcdf.

The resulting enrichment scores were used as features for downstream machine-learning analysis.

#### Model fitting and prediction

Disease classification was performed using a random forest (RF) ensemble machine learning model implemented in the tidymodels framework (v1.2.0). Model tuning was performed using 10-fold cross-validation with three repeats. The RF hyperparameters optimized during cross-validation included the number of features considered at each split node and the minimum number of samples required to split a node. The hyperparameter configuration yielding the highest cross-validation accuracy was selected for the final model. The optimized RF model was then trained on the full training dataset and subsequently applied to independent testing datasets to generate disease classification predictions. Model performance was evaluated using accuracy and the area under the receiver operating characteristic curve (ROC–AUC). ROC curves were generated using the pROC R package (v1.19.0.1) [19].

#### Model benchmarking

To evaluate the performance of WBT-DC, we benchmarked it against conventional models that directly use gene expression values as input features. For a fair comparison, both approaches were applied to the same datasets with an equal number of features. Specifically, the conventional models used the top 100 upregulated and 100 downregulated DEGs identified from the training datasets. The RF model architecture and hyperparameter tuning procedures were kept identical to those used in the WBT-DC pipeline to ensure consistency.

This study included datasets from four diseases: CD, UC, ALS, and RA. Datasets for CD, UC, and ALS were obtained from publicly available repositories, with each disease comprising at least one independent testing cohort and covering both RNA-seq and microarray platforms. For RA, the training dataset consisted of in-house RNA-seq data, while an independent public microarray dataset was used for validation.

In the machine-learning–based WBT-DC pipeline, gene expression profiles were transformed into gene set enrichment scores using a rank-based strategy. DEGs between disease and healthy samples were first identified in the training datasets using an adjusted P value < 0.05. From the top-ranked DEGs (ordered by log₂ fold change), 100 upregulated and 100 downregulated gene sets were randomly selected. The optimal gene set size was determined using the Wilcoxon rank-sum test by assessing differences in GSVA enrichment scores between disease and control samples across multiple gene set size intervals. GSVA enrichment scores were subsequently calculated using VST-normalized counts for RNA-seq data and log₂-transformed expression values for microarray data.

Disease classification was performed using RF models, with hyperparameters optimized via 10-fold cross-validation with three repeats. The optimized models were then applied to independent testing datasets to evaluate classification performance.

## Results

### WBT-DC pipeline for disease classification using WBT data

To evaluate the predictive performance of the WBT-DC pipeline, we applied it to datasets from CD, UC, and ALS. Sample sizes and details of each dataset are summarized in Table 1.

**Fig. 2:**
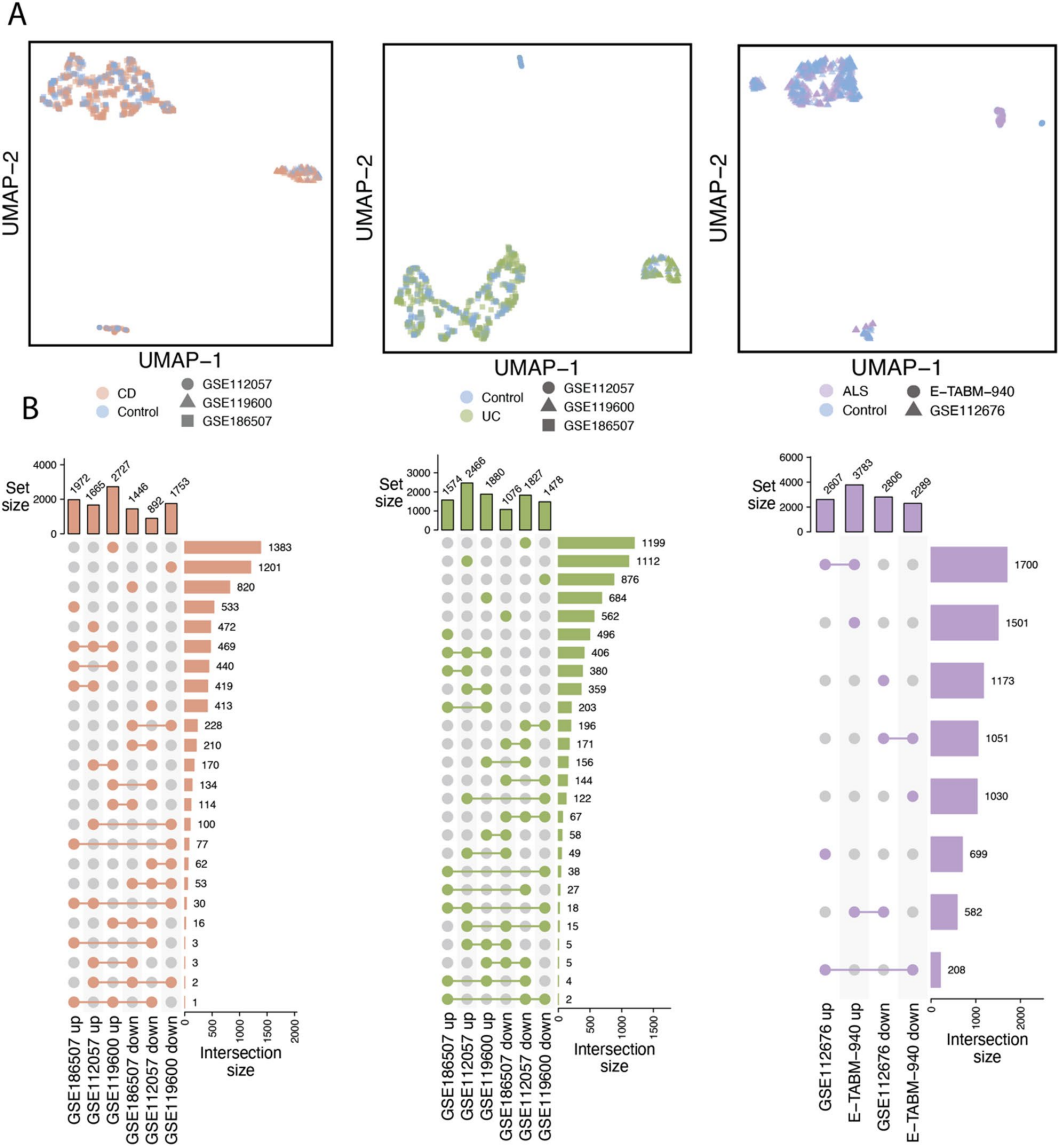
Gene expression profiles across disease datasets. **A**) UMAP plots displaying gene expression profiles across publicly available datasets for CD, UC, and ALS. Three independent cohorts were included for both CD and UC, and two independent cohorts were included for ALS. **B**) UpSet plots illustrating the intersection sizes of DEGs across cohorts within each disease group

We first examined the global characteristics of gene expression profiles across cohorts. UMAP analysis revealed that samples clustered primarily by cohort rather than disease status, indicating pronounced batch effects across datasets within the same disease context (Fig. 2A). Consistently, UpSet plots showed limited overlap among DEGs identified across cohorts (Fig. 2B). Together, these observations highlight substantial heterogeneity among datasets and underscore the need for robust feature summarization strategies to enable reliable disease classification.

**Fig. 3:**
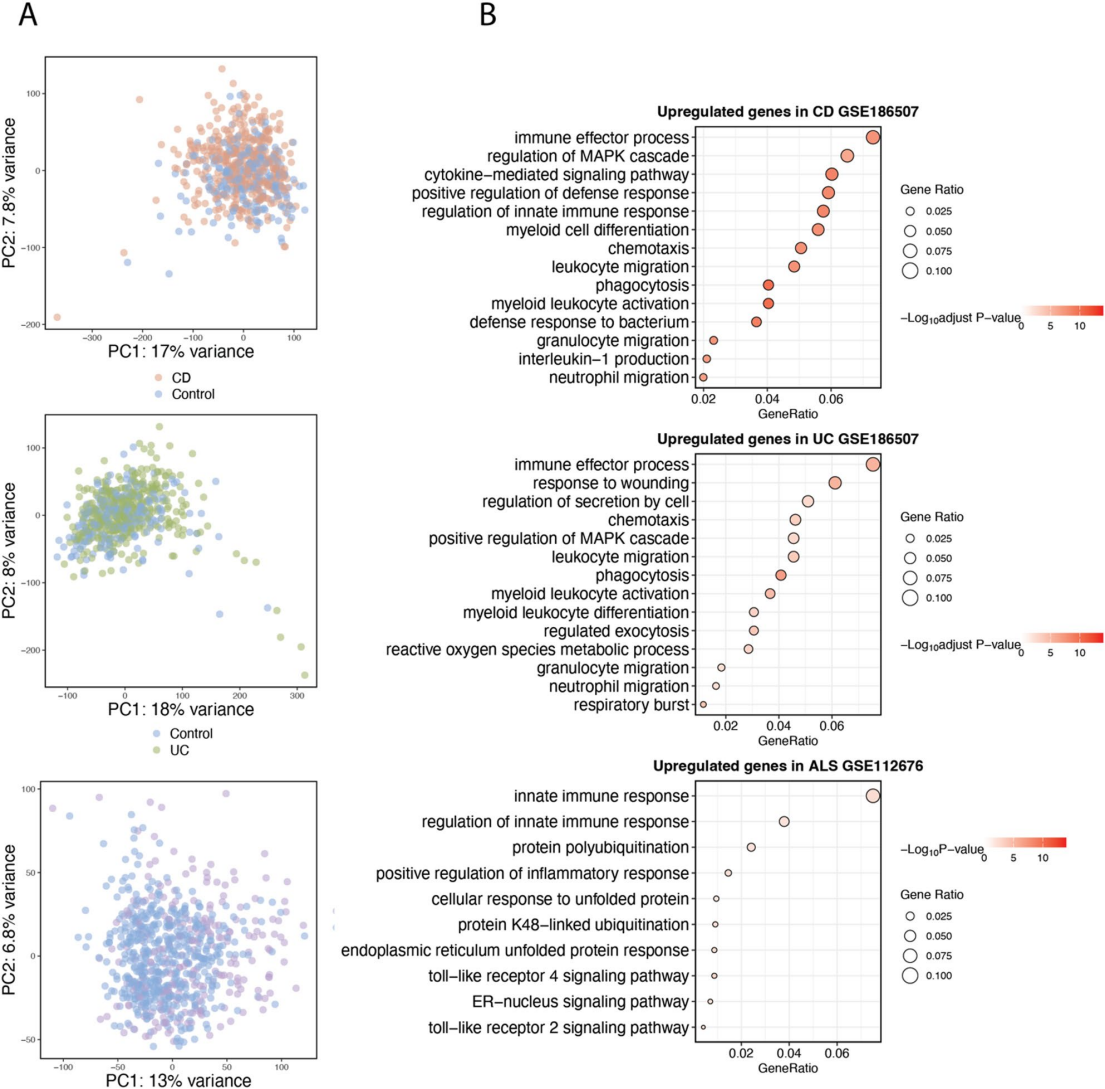
Gene expression patterns and pathway enrichment in training datasets. (**A**) PCA plots showing gene expression profiles of disease and healthy samples in the training datasets for CD, UC, and ALS. (**B**) Dot plots summarizing GSOA results for CD, UC, and ALS. Dot size represents the gene ratio (the proportion of DEGs assigned to each pathway relative to the total number of DEGs analyzed

For each disease, the dataset with the largest sample size was selected as the training cohort: 429 CD cases and 209 controls (GSE186507, RNA-seq), 378 UC cases and 209 controls (GSE186507, RNA-seq), and 233 ALS cases with 508 controls (GSE112676, Illumina microarray). Principal component analysis (PCA) indicated that disease and control samples in the training datasets were not clearly separated along the first two principal components (Fig. 3A), reflecting the complexity of transcriptomic signals in whole blood. GSOA demonstrated that DEGs were enriched in pathways consistent with known disease biology. Specifically, upregulated genes in CD were enriched in pathways related to innate immune activation, leukocyte migration, and chemotaxis. In UC, enrichment was primarily observed in neutrophil-driven inflammatory pathways and reactive oxygen species production. In ALS, enriched pathways included Toll-like receptor signaling, endoplasmic reticulum stress, and protein ubiquitination, reflecting neuroinflammation and disrupted protein homeostasis (Fig. 3B). Besides, similar biological processes were consistently observed in the independent testing dataset (Supplementary Fig. 3).

**Fig. 4:**
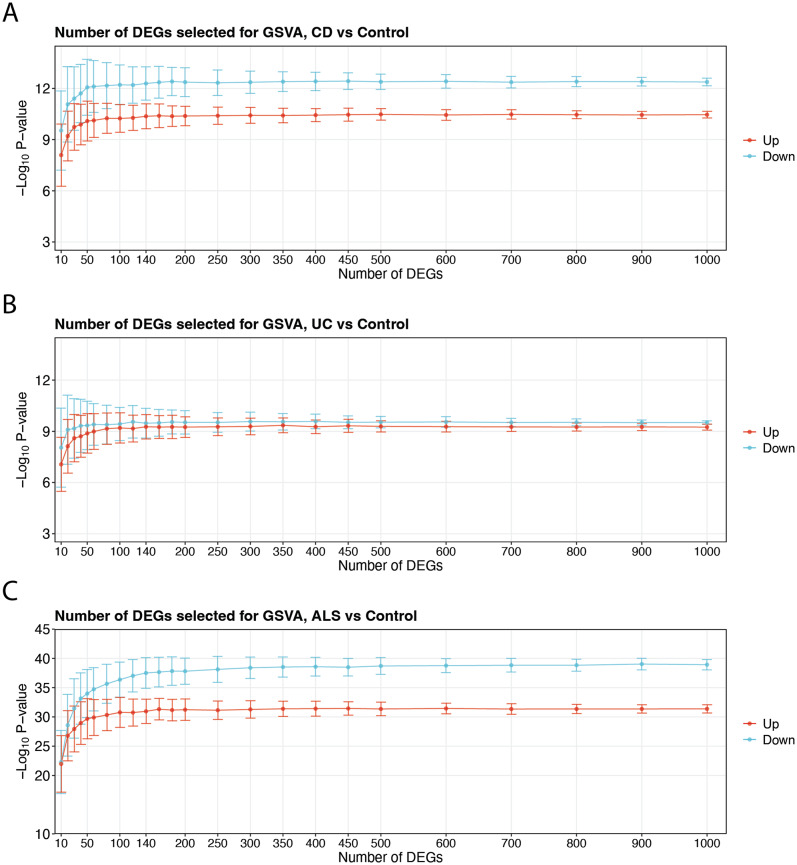
Statistical significance of DEG-based enrichment across gene set sizes. Curve plots displaying the significance of differences in gene set enrichment scores between disease and healthy samples across varying gene set size intervals in CD (**A**), UC (**B**), and ALS (**C**)

Next, GSVA was applied to extract robust features from the training datasets. Figure 4 shows the differences in GSVA enrichment scores between disease and control samples across varying gene set sizes. Stabilization of enrichment score differences was used to determine optimal gene set sizes. Optimal performance was observed with gene sets containing 60–100 genes for CD, 30–50 genes for UC, and 50–100 genes for ALS. From the top-ranked DEGs, we randomly generated 100 upregulated and 100 downregulated gene sets for each disease. GSVA enrichment scores derived from these 200 gene sets were subsequently used as features to train the machine-learning models.

**Table 1:** Disease cohorts analyzed in this study

Diseases	Dataset	Male %	Age (Mean ± SD)	Sample number (Disease: Control)	Platform
Crohn’s disease (CD)	GSE186507	339 (53.1%)	45.3 ± 14.8	429:209	RNA sequencing: Illumina HiSeq 2500
	GSE112057	36 (60.0%)	14.1 ± 3.9	60:12	RNA sequencing: Illumina HiSeq 2000
	GSE119600	/	/	95:47	Microarray: Illumina HumanHT-12 V4.0 expression beadchip
Ulcerative Colitis (UC)	GSE186507	311 (53.0%)	48.2 ± 15.1	378:209	RNA sequencing: Illumina HiSeq 2500
	GSE112057	8 (53.3%)	15.2 ± 5.8	15:12	RNA sequencing: Illumina HiSeq 2000
	GSE119600	/	/	93:47	Microarray: Illumina HumanHT-12 V4.0 expression beadchip
Amyotrophic Lateral Sclerosis (ALS)	GSE112676	421 (56.8%)	62.4 ± 11.8	233:508	Microarray: Illumina HumanHT-12 V3.0 expression beadchip
	E-TABM-940	47 (55.3%)	53.4 ± 12.7	57:28	Microarray: Affymetrix GeneChip Human Genome U133 Plus 2.0
Rheumatoid Arthritis (RA)	In-house data	77 (46.7%)	44.8 ± 14.5	61:104	RNA sequencing: Illumina NovaSeq6000
	GSE93272	17 (13.8%)	52.0 ± 14.9	80:43	Microarray: Affymetrix Human Genome U133 Plus 2.0 Array

### Single-platform evaluation of the WBT-DC pipeline

**Fig. 5:**
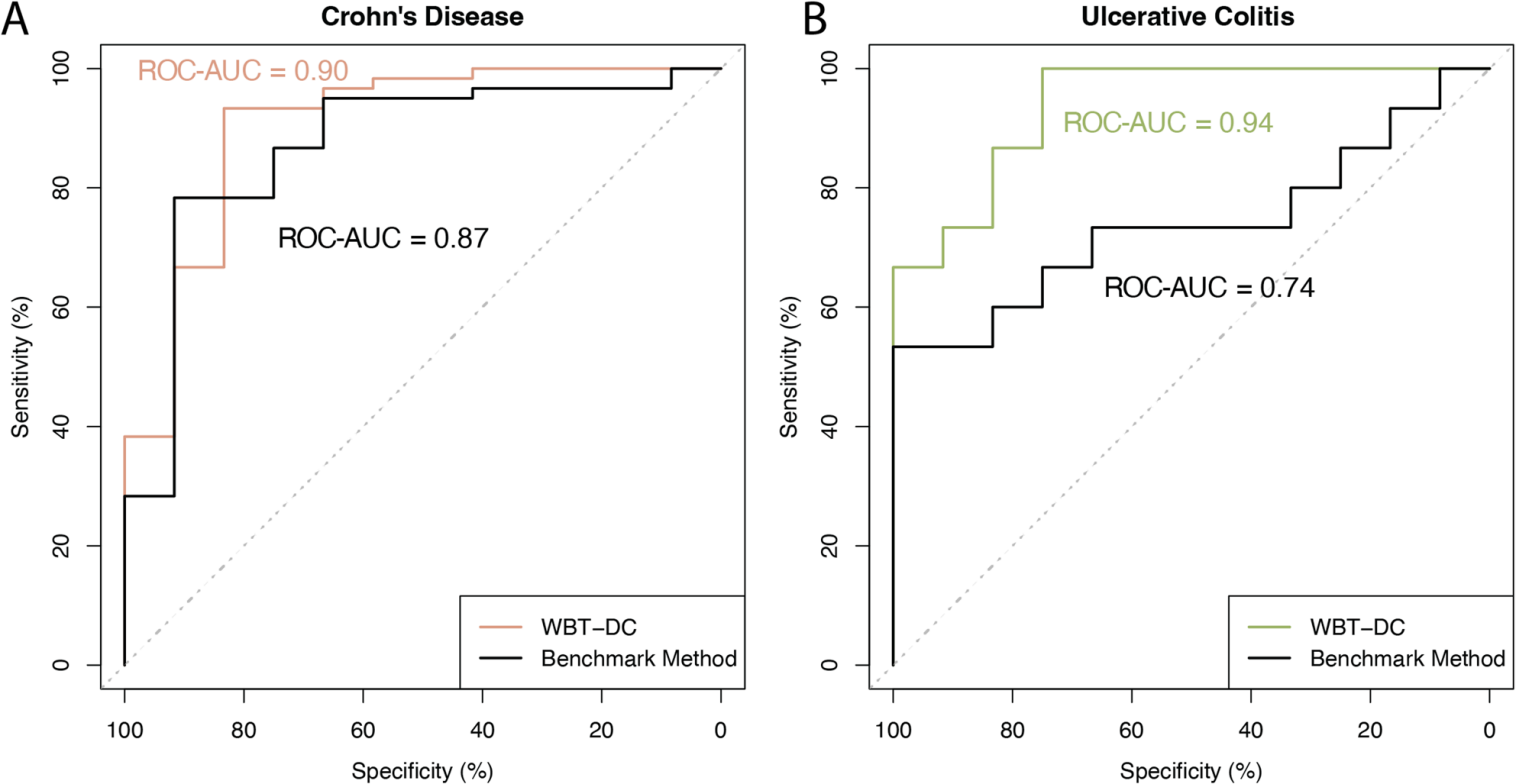
Performance of the WBT-DC pipeline using single-platform whole-blood transcriptomic data

We first evaluated model performance using independent cohorts in which both the training and testing datasets were generated on the same transcriptomic platform. For CD and UC, RNA-sequencing data from GSE186507 were used for training, and GSE112057 served as the independent testing dataset. Features were extracted by computing GSVA enrichment scores for 200 gene sets derived from the training data and subsequently used as inputs for disease classification models. Using this pipeline, the model achieved a balanced accuracy of 0.79 and an ROC–AUC of 0.90 for CD, and a balanced accuracy of 0.82 and an ROC–AUC of 0.94 for UC (Fig. 5A, B), respectively.

We further evaluated model performance using conventional approaches that directly employed gene expression values of the top 100 upregulated and downregulated DEGs from the training datasets as features. This method showed reduced performance on the same independent testing datasets compared with WBT-DC, achieving a balanced accuracy of 0.50 and an ROC–AUC of 0.87 for CD, and a balanced accuracy of 0.50 and an ROC–AUC of 0.74 for UC.

ROC curves showing model performance when both training and testing datasets were generated using the same transcriptomic platform. RNA-sequencing data from GSE186507 were used for model training, and GSE112057 served as the independent testing dataset for both CD and UC. Colored curves represent the performance of the WBT-DC models, whereas black curves denote conventional models based directly on gene expression values.

### Cross-platform evaluation of the WBT-DC pipeline

We next evaluated model performance in cross-platform settings, where training and testing datasets were generated using different transcriptomic platforms. For CD and UC, models were trained on RNA-sequencing data (GSE186507) and subsequently evaluated on an independent Illumina microarray dataset (GSE119600) for validation. For ALS, the disease classification model was trained on GSE112676 (Illumina microarray) using the same pipeline and tested on E-TABM-940 (Affymetrix microarray).

**Fig. 6:**
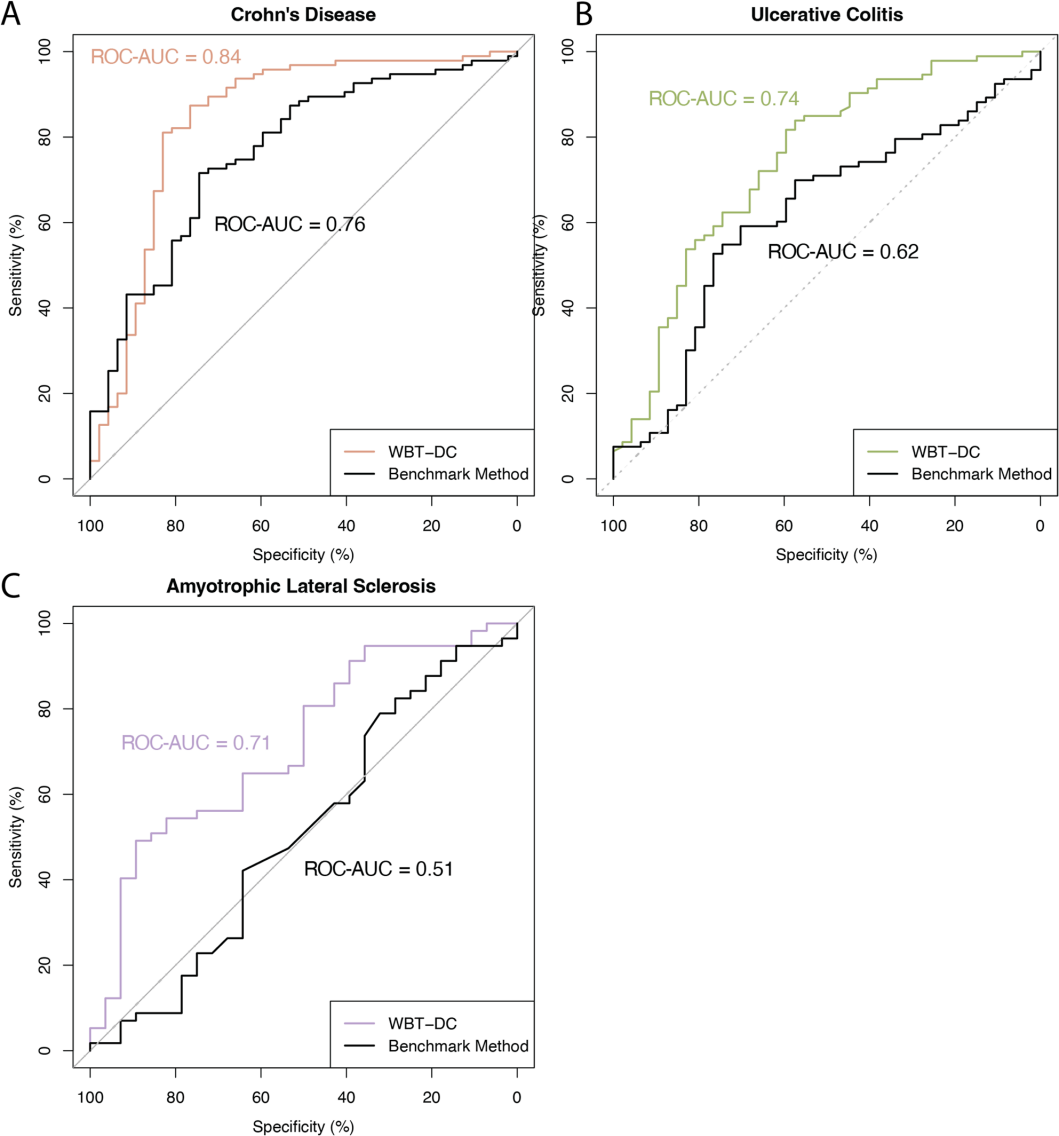
Performance of the WBT-DC pipeline in cross-platform evaluations

Under these cross-platform evaluations, the models achieved an accuracy of 0.82 and an ROC–AUC of 0.84 for CD, an accuracy of 0.74 and an ROC–AUC of 0.74 for UC, and a balanced accuracy of 0.67 and an ROC–AUC of 0.71 for ALS, as shown in Fig. 6C. In contrast, conventional models based directly on top gene expression values showed lower classification performance, with an accuracy of 0.67 and an ROC–AUC of 0.76 for CD, an accuracy of 0.66 and an ROC–AUC of 0.62 for UC, and a balanced accuracy of 0.53 and an ROC–AUC of 0.51 for ALS. Overall, these results demonstrate that our models maintain robust performance across different transcriptomic platforms.

ROC curves showing model performance when training and testing datasets were generated using different transcriptomic platforms. For CD and UC, RNA-sequencing data from GSE186507 were used for model training, and an independent Illumina microarray dataset (GSE119600) was used for testing. For ALS, the model was trained on GSE112676 (Illumina microarray) and tested on E-TABM-940 (Affymetrix microarray). Colored curves represent the performance of the WBT-DC models, whereas black curves denote conventional models based directly on gene expression values. Overall, WBT-DC achieved higher ROC–AUC values across all disease cohorts compared with conventional approaches (CD: 0.84 vs. 0.76; UC: 0.74 vs. 0.62; ALS: 0.71 vs. 0.51).

### Application of the WBT-DC pipeline to an internal rheumatoid arthritis cohort

Our cross-platform WBT-DC pipeline demonstrated robust performance on publicly available datasets. We next recruited an internal RA cohort comprising 61 patients and 104 healthy controls and applied the WBT-DC pipeline to this cohort to demonstrate its practical applicability. In addition, a publicly available Affymetrix microarray dataset (GSE93272), comprising 80 RA samples and 43 healthy controls, was used as an independent testing cohort.

**Fig. 7:**
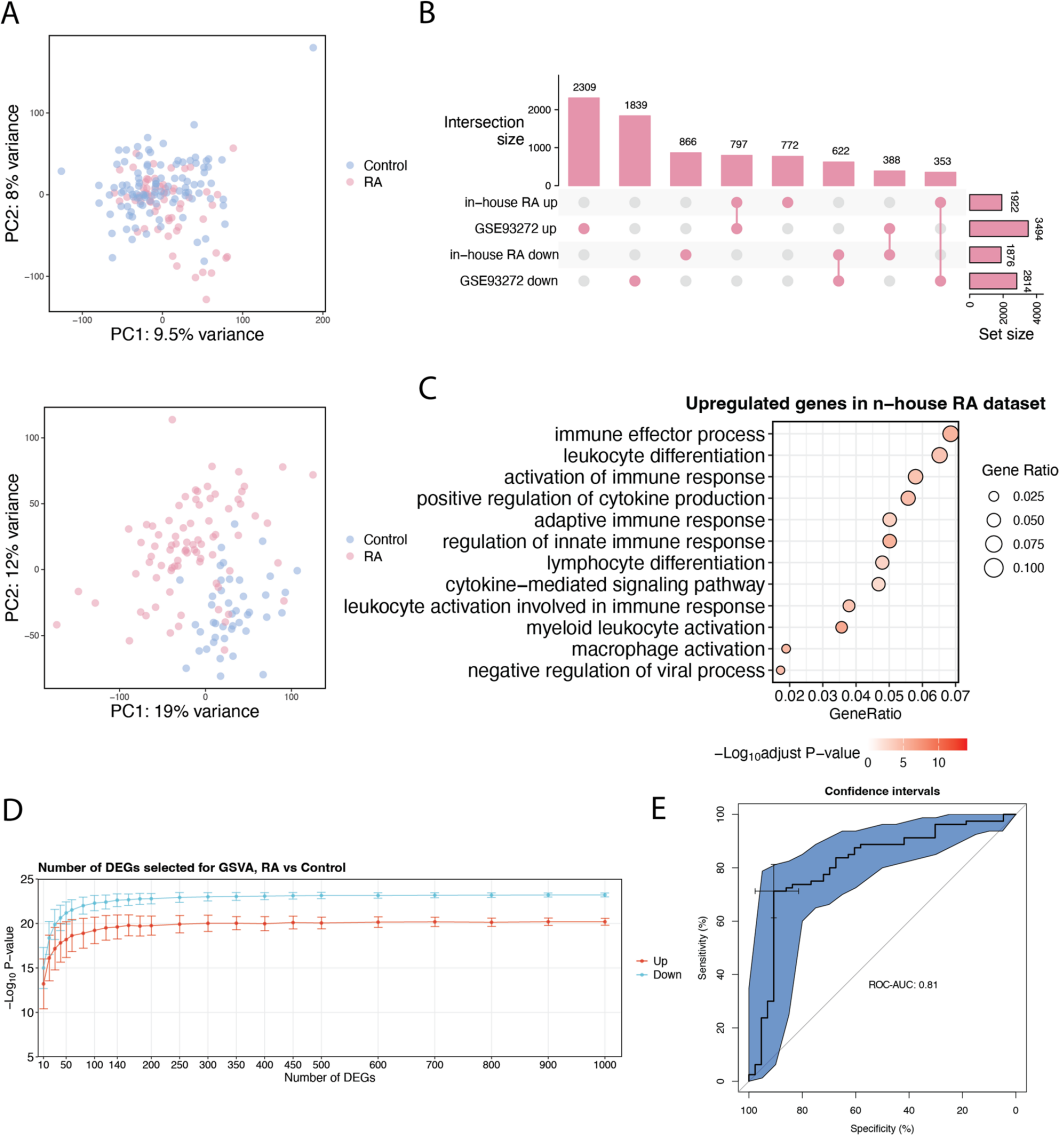
Application of the WBT-DC pipeline to a RA cohort. (**A**) PCA plots showing gene expression profiles of the in-house RA training cohort (upper panel) and the independent Affymetrix microarray testing cohort (GSE93272, lower panel). (**B**) UpSet plots illustrating the intersection size of DEGs between training and testing datasets. (**C**) Dot plots showing the results of GSOA in the training dataset. (**D**) Curve plots showing the statistical significance of differences in GSVA enrichment scores between RA and healthy samples across varying gene set sizes in the training dataset. (**E**) ROC and calibration curves evaluating the classification performance of the WBT-DC model in the RA cohort

PCA revealed distinct gene expression patterns between the two cohorts (Fig. 7A). In the in-house RA training cohort, we identified 1,922 upregulated and 1,876 downregulated genes (Fig. 7B). Functional enrichment analysis showed that the upregulated genes were predominantly enriched in immune response–related pathways, including immune effector processes, leukocyte differentiation, and cytokine production (Fig. 7C). Using the Wilcoxon rank-sum test, we observed that differences in GSVA enrichment scores between RA and healthy samples stabilized when gene set sizes ranged from 60 to 100 genes. Accordingly, gene sets were randomly selected from the top-ranked 200 upregulated and downregulated DEGs in the training data. GSVA enrichment scores derived from these gene sets were calculated for both training and testing datasets and used as features for machine-learning model construction. When evaluated on the independent testing dataset, the model achieved a balanced accuracy of 0.78 and an ROC–AUC of 0.81 (Fig. 7E). Together, these results highlight the robustness and practical applicability of the WBT-DC pipeline for disease classification.

## Discussion

In this study, we developed a machine learning–based whole-blood transcriptomics disease classification (WBT-DC) pipeline for disease prediction using WBT data. The pipeline demonstrated robust classification performance across multiple datasets and transcriptomic platforms. We first validated the model using publicly available datasets from three distinct diseases: CD and UC, which are associated with digestive system dysfunction, and ALS, a neurological disorder. We then extended the application of the WBT-DC pipeline to in-house RNA-sequencing data from an RA cohort, representing an autoimmune disease affecting the joints. Across these diverse disease contexts, the WBT-DC pipeline consistently achieved strong predictive performance, highlighting its robustness and broad applicability.

It is well established that complex diseases are driven by transcriptional dysregulation across multiple tissues [20–22]. Given its intimate connection to the entire body, the circulatory system provides a unique window into systemic biological states, raising the question of how effectively WBT captures tissue-specific transcriptional features. A recent study demonstrated that WBT data can predict tissue-specific expression levels for approximately 60% of genes across 32 tissues [3], making its potential as a powerful medium for monitoring systemic diseases. Our results further demonstrated that DEGs identified from whole-blood transcriptomic data between disease and healthy individuals were significantly enriched in pathways closely related to disease mechanisms. This enrichment was observed not only in immune-mediated diseases such as CD, UC, and RA, but also in ALS, a motor neuron disease.

Machine learning has been widely used in biomarker-based disease prediction and has shown great potential [23]. Transcriptomic data have made significant progress in identifying risk-associated biomarkers [24]. Early studies primarily focused on single-gene biomarkers. However, recent work has demonstrated that multi-gene signatures provide improved robustness and predictive performance [25]. WBT has recently emerged as a powerful approach for disease prediction [26]. However, a key challenge in WBT-based machine learning studies is mitigating batch effects arising from the integration of transcriptomic data generated across different datasets and platforms. In this study, we addressed this issue by implementing a rank-based feature extraction strategy that transforms gene expression values into gene set enrichment scores. This transformation places features on a comparable scale across datasets, thereby reducing platform-specific biases. Our approach emphasizes the most informative DEGs from the training data by repeatedly sampling from top-ranked DEGs to construct gene sets for GSVA computation. Specifically, for CD cohorts, the enriched pathways were predominantly associated with innate immune activation and host–microbial interactions. These findings are consistent with the well-established role of dysregulated immune responses to intestinal microbiota in disease pathogenesis [27]. In ALS, enriched pathways were primarily associated with Toll-like receptor signaling, cellular responses to unfolded proteins, and leukocyte migration and chemotaxis. These findings suggest a coordinated pathological process involving innate immune activation, disrupted protein homeostasis, and systemic immune involvement [28, 29]. In RA, enriched pathways were mainly associated with immune activation, including myeloid leukocyte and macrophage activation, as well as regulation of innate immune responses. Enrichment of cytokine-mediated signaling and positive regulation of cytokine production highlights the central role of pro-inflammatory cytokines. In addition, pathways related to adaptive immune responses and lymphocyte differentiation suggest the involvement of T and B cells in sustaining chronic inflammation [30]. These results suggest that the features captured by our pipeline reflect biologically relevant processes associated with each disease.

This study has several limitations. First, the current pipeline relies on aggregated gene set features and therefore may not explicitly identify individual genes that play dominant roles in disease classification. Second, while our approach was evaluated across seven independent datasets spanning various disease categories, further validation is required through larger cohorts and a broader spectrum of conditions, such as cancer and metabolic disorders. Third, to facilitate clinical translation of whole-blood transcriptomics–based machine-learning models, the development of user-friendly interfaces and deployable platforms will be necessary to enable widespread adoption in routine clinical practice.

In conclusion, we developed a novel machine learning–based pipeline for disease classification using whole-blood transcriptomic data that overcomes key limitations associated with invasive tissue sampling. The WBT-DC pipeline demonstrated robust performance across multiple transcriptomic platforms by effectively mitigating batch effects. We validated its performance using diverse public datasets and further demonstrated its applicability through a case study based on an in-house rheumatoid arthritis cohort. The consistent and robust performance of WBT-DC highlights its potential for future clinical applications in disease classification and precision medicine.

## Supplementary Information

Below is the link to the electronic supplementary material.

Supplementary Material 1

## Data Availability

Publicly available data can be accessed from databases using their respective reference numbers. The dataset for rheumatoid arthritis is available in the GEO database (GSE282218). All code necessary for the analysis and visualization is available at: https://github.com/Mengzhensw/WBT-DC-Pipeline-Whole-Blood-Transcriptomics-data-based-Disease-Classification.
