# Supplementary material for: WBT-DC pipeline: a cross-cohort and cross-platform disease classification pipeline based on whole-blood transcriptomics
Source: J Transl Med. 2026 May 11;24:679. doi: 10.1186/s12967-026-08254-3 (PMC13173921; doi:10.1186/s12967-026-08254-3)
Supplement: Supplementary file 1 — Supplementary Material 1 [file 12967_2026_8254_MOESM1_ESM.docx]

**Supplementary Figure 1**. Violin plot showing the expression levels of the top 10 highest expressed genes in red blood cells after log10 transformation.

**
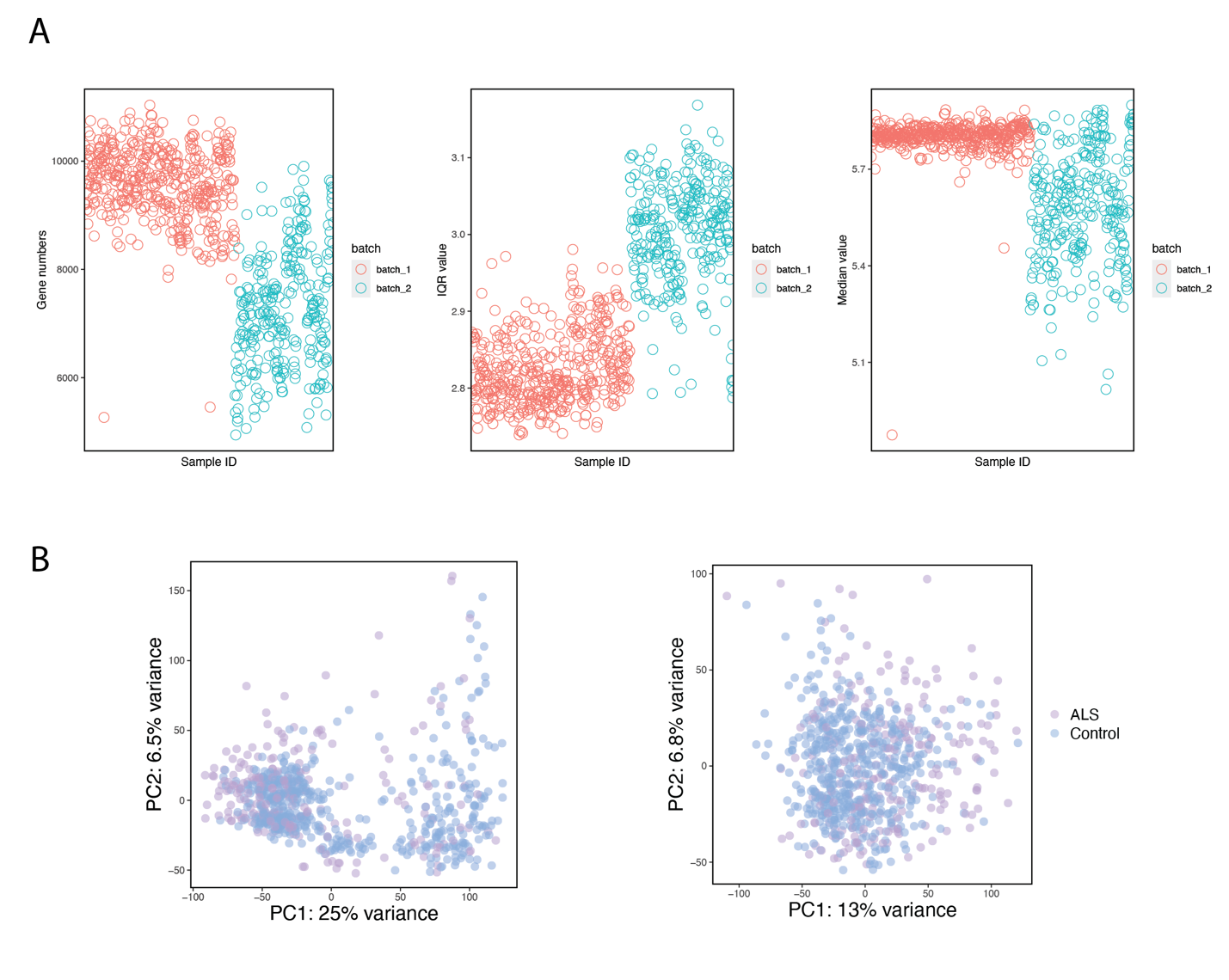
**

**Supplementary Figure 2**. Batch effect in GSE112676 dataset

1. A batch effect was identified, characterized by differences in signal interquartile range (IQR), median intensity, and the number of detected protein-coding genes between the first set of 448 samples and the second set of 293 samples
2. PCA plots before and after batch effect removal using combat.


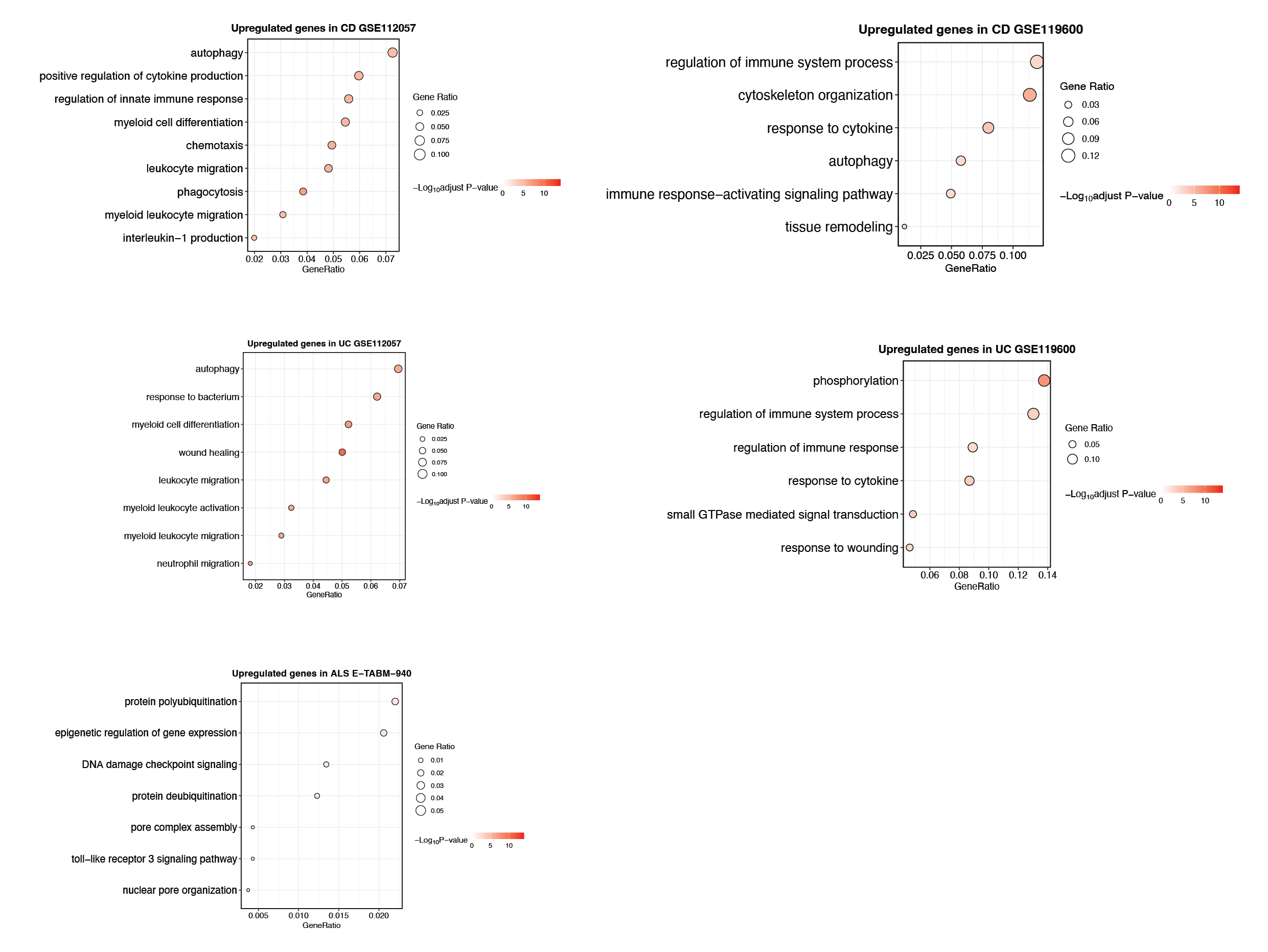


**Supplementary Figure 3**. GSOA results in the testing datasets for CD, UC, and ALS.
